# Decompressive cervical laminectomy and lateral mass screw-rod arthrodesis. Surgical analysis and outcome

**DOI:** 10.1186/1748-7161-6-10

**Published:** 2011-05-19

**Authors:** Moh'd M Al Barbarawi, Ziad A Audat, Moutasem M Obeidat, Tareq M Qudsieh, Waleed F Dabbas, Mouness H Obaidat, Anas A Malkawi

**Affiliations:** 1 Department of Neuroscience/ Division of Neurosurgery, Level 7A. King Abdullah University Hospital, Jordan University of Science and Technology, Irbid-Amman Street. P.O.box 3030, Irbid, Jordan; 2 Department of Orthopeadic, Level 8A. King Abdullah University Hospital, Jordan University of Science and Technology, Irbid-Amman Street, P.O.box 3030, Irbid, Jordan

**Keywords:** lateral mass, arthrodesis, cervical myelopathy, spinal fixation, decompressive laminectomy

## Abstract

**Background:**

This study evaluates the outcome and complications of decompressive cervical Laminectomy and lateral mass screw fixation in 110 cases treated for variable cervical spine pathologies that included; degenerative disease, trauma, neoplasms, metabolic-inflammatory disorders and congenital anomalies.

**Methods:**

A retrospective review of total 785 lateral mass screws were placed in patients ages 16-68 years (40 females and 70 males). All cases were performed with a polyaxial screw-rod construct and screws were placed by using Anderson-Sekhon trajectory. Most patients had 12-14-mm length and 3.5 mm diameter screws placed for subaxial and 28-30 for C1 lateral mass. Screw location was assessed by post operative plain x-ray and computed tomography can (CT), besides that; the facet joint, nerve root foramen and foramen transversarium violation were also appraised.

**Results:**

No patients experienced neural or vascular injury as a result of screw position. Only one patient needed screw repositioning. Six patients experienced superficial wound infection. Fifteen patients had pain around the shoulder of C5 distribution that subsided over the time. No patients developed screw pullouts or symptomatic adjacent segment disease within the period of follow up.

**Conclusion:**

decompressive cervical spine laminectomy and Lateral mass screw stabilization is a technique that can be used for a variety of cervical spine pathologies with safety and efficiency.

## Introduction

Posterior cervical fixation with lateral mass screws was first introduced by Roy-Camille in 1979; it has been increasingly used since that time to treat a wide range of cervical spine disorders [1]. posterior cervical fixation was frequently involved in form of wire and bone construct fixation. With a proven long-term effectiveness, and requires no special skills or x-ray guidance [2-4]. posterior cervical wire fixation may not be efficient in osteoporotic patient, as this technique can compromise the posterior cervical elements and may result in aggravating the primary pathology and worsen up the neurological status that requires full fixation by using the lateral mass fixation technique [5-7]. Furthermore, Stainless-steel wire can interfere with postoperative magnetic resonance (MR) imaging results, in contrast to the MRI compatible titanium screw/rod constructs. Lateral mass screw fixation has advantages over standard posterior wiring techniques; it can be done easily for many levels on patients with laminectomy and it can preserve the biomechanical forces. However, serious neural or vascular injury can explain the reservations of unfamiliar surgeons to this practice. so far, this method shows a global acceptance by many surgeons [8-10].

In this communication we reviewed and analyzed the surgical outcome of 110 consecutive cases treated with decompressive cervical Laminectomy and lateral mass fixation by using Anderson - Sekhon technique to deal with a punch of cervical disorders. Besides Operative and clinical outcomes; post operative computed tomography (CT) analysis is also provided with particular emphasis on clinical improvement and neurologic and vascular complications.

## Statistical methods

The Statistical Package for Social Sciences software (SPSS, version 15) was used for data processing and analysis. The subjects' variables were described using frequency distribution for categorical variables and mean and standard deviation for continuous variables. P value of ≤ 0.005 is considered not significant.

## Clinical materials and methods

The study was approved by the ethical committee for human research (IRB) at Jordan University of Science and Technology. The study group consisted of 110 patients treated for multiple cervical pathologies performed in king Abdullah university hospital between Dec, 2005 and January, 2011. Decompressive cervical Laminectomy with a total of 785 lateral mass screws was applied in different cervical spine levels to deal with degenerative disease, spinal trauma (fracture-dislocation and hyperextension spinal cord injury ), cervical spine neoplasms, rheumatoid arthritis, calcification of the posterior longitudinal ligament and congenital anomalies. Exclusionary criteria included; Patients with soft tissue spinal cord tumours not affecting the bony elements or spinal instability, chronic or active infection. The severity of cervical myelopathy was assessed by using Nurick scale [11]. The patient demographics were reviewed and analyzed in a retrospective manner.

### Surgical Technique

The surgery was performed in a steady fashion. Fiberoptic Intubation was used as indicated. All cases were performed with digital fluoroscopic guidance. The lateral masses were initially drilled and tapped prior to laminectomy. Placement of screws was performed after cervical decompression. The entry point was about 1 mm medial to the midpoint of the lateral mass. The screws were angulated about 25^0 ^laterally and superiorly to achieve the best position of the lateral mass and to minimize the risk of neural or vascular violation, (modified Anderson and Sekhon techniques) [8,12]. At C7 level, when its lateral mass is included in the fixation a more angulation was affected in comparison with other trajectories.

Intraoperatively, each screw position was assessed separately by imaging guidance before the final placement. For biological fusion; chips of auto-graft bone from the posterior elements or artificial bone were placed over the decorticated lateral masses and into the appropriate facet joints after screw insertion. Postoperatively all patient were placed into a hard nock collar and plain x-ray done on the first post operative day.

**Figure 1:**
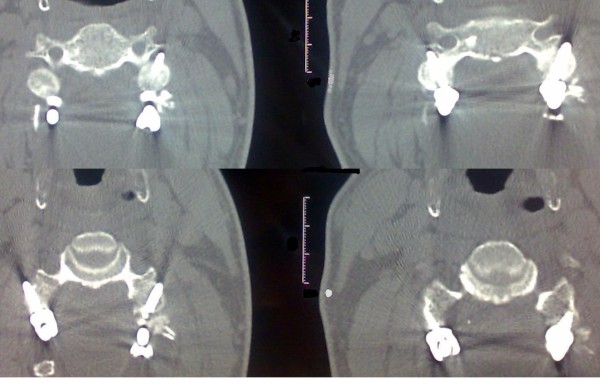
**Postoperative axial CT scan slice of C5 showing typical bicorticate screw place in the body of the lateral mass with a 14 mm length**. The vertebral foramen is seen not violated. Some bony fusion is also observed (arrow)

Any intraoperative or postoperative clinical or radiological evidence of nerve root or vertebral artery violation were also evaluated immediately by considering a thin-slice CT scan to evaluate all lateral mass screws position, encroachment into the foramen transversarium or into the neural foramen (figure 1).

Postoperatively patients were evaluated clinically and radiologically at 4 weeks, 2 months, 6 months, 12 months, 24 months and 36 months. Follow-ups, in this study, ranged from 2 months to 3 years. All myelopathic patients discharged for rehabilitation programme.

## Results

The demographics of the 785 lateral mass screws per 110 patients are shown in (Table 1). The majority of patients were males, with an average age of 16-45 years. Some co morbidities were encountered and managed adequately.

**Table 1:** shows the demographic distribution and the indications for decompressive Laminectomy and lateral mass fixation

Patient Demographics: (number = 110)	Number	%
**Males**	70	64

**Females**	40	36

**Age range**	16-68	

Average	44.8	

**Indication:**		

Degenerative disease	73	66.3

Cervical spine trauma		20.9

Fracture- dislocation	10	

Central cord syndrome	13	

Neoplastic lesions	4	3.7

Metabolic or inflammatory disorders	4	3.7

Congenital anomalies	6	5.4

**Levels included:**		

C3-6	39	35.5

C3-5	9	8.3

C4-6	38	34.5

C3-7	10	9.2

C4-7	4	3.7

C5-7	3	2.8

Cranio-cervical	**6**	**5.6**

**Cervicothoracic incorporation**	**1**	**0.9**

Most cases were performed for degenerative spondylotic myelopathy and trauma. The majority were stand-alone subaxial fixations.

**Figure 2:**
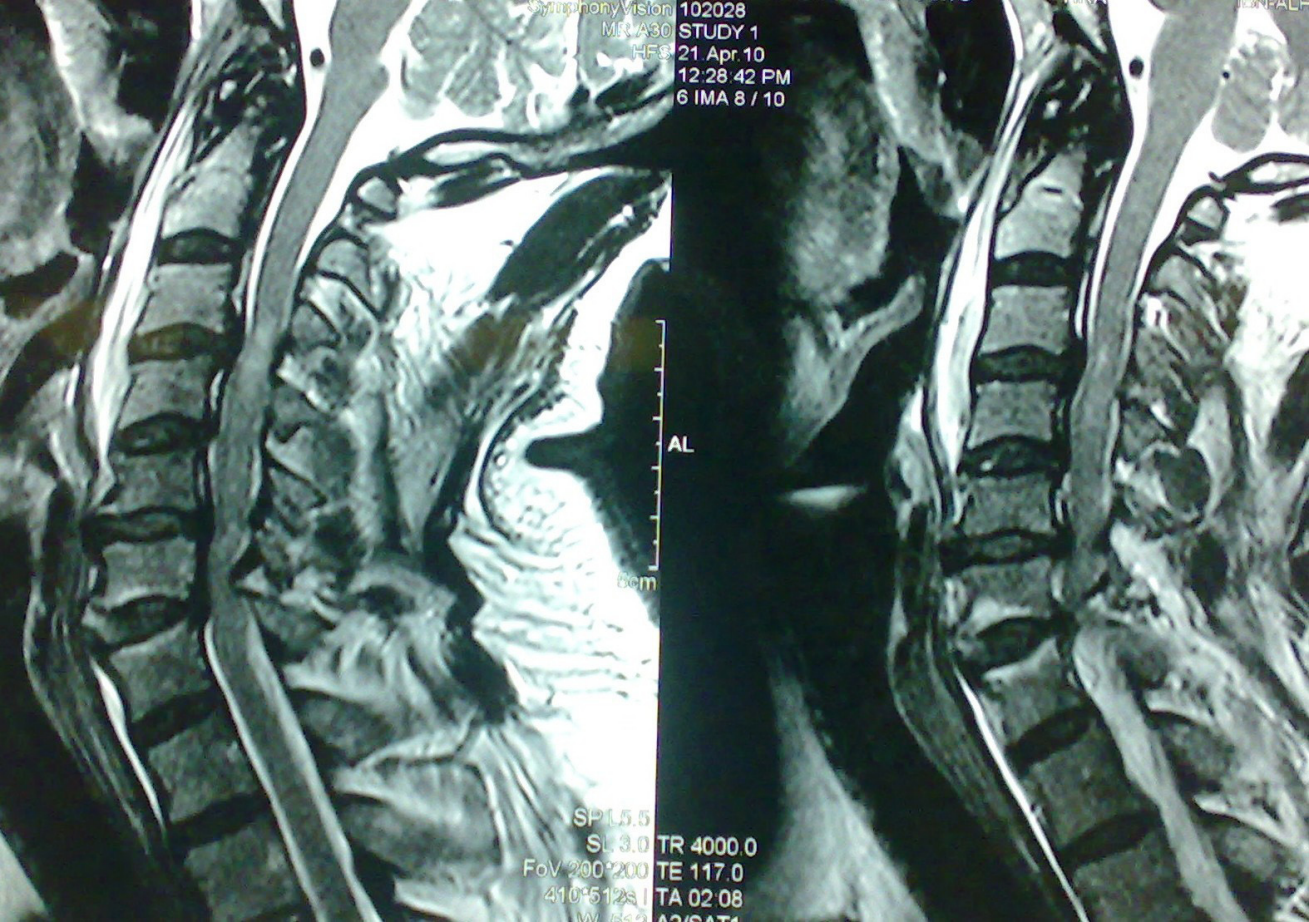
**Pre operative sagittal MRI T2W**. Reveals severe cervical spine spondylotic changes with a spinal cord signal at C3/4 level in a patient of a 65 years old with traumatic spinal cord injury.

**Figure 3:**
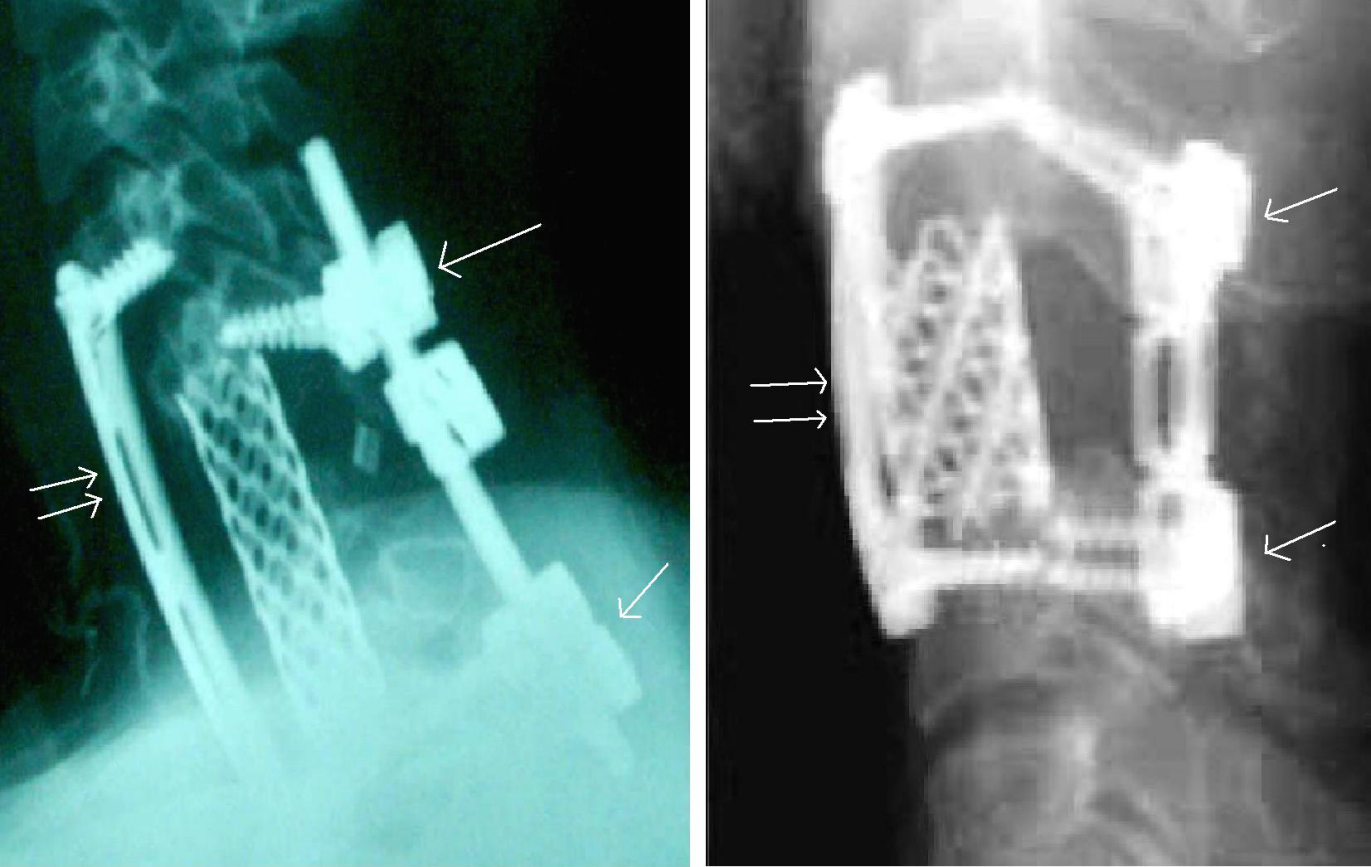
**Post operative lateral X-rays**. Anterior and posterior spinal approaches with corpectomy and total resection of the spinal tumor from front and back in a staged operation with lateral mass fixation.

The 110 cases included in this report covered different pathologies. The indications included symptomatic degenerative cervical spine disease (severe cervical spine spondylosis) (73 cases), cervical spine injury (23 cases; 10 with fracture- sublaxation and 13 with central cord syndrome) (figure 2), cervical spinal/vertebral body neoplastic lesions (4 cases), idiopathic calcification of the posterior longitudinal ligament (2 patients), rheumatoid arthritis (2 cases), congenital anomalies such as; platy basia, assimilation of C1, klipple feil syndrome, achondroplasia (6 patients). A variety of different implants were used including Vertex (Medtronic Sofamor-Danek) and Oasys (Stryker Spine, France) polyaxial screw/rod constructs. All polyaxial screw-rod constructs were affected adequately in the subaxial region and CI. Screws of 12-14 mm length and 3.5 mm width were usually used for fixation in the majority of cases and 28-30 mm length when used for C1. The majority of constructs were stand-alone subaxial constructs; cross link was used in most of cases. However, in certain cases the lateral mass fixation was also incorporated as part of an occipitocervical or cervicothoracic fusion or as additional reinforcement for an anterior constructs (Figure 3).

Intraoperatively, of the 785 lateral mass screws placed, there was no observation of vertebral artery injury or nerve root damage. Durotomy occurred in five cases that required intraoperative repair; all of those patients had a significant cervical stenosis. C7 was able to be adequately drilled with a steeper trajectory in seven cases. Poor screw placement occurred in about 14 screws from lateral mass breakout in patients with osteoporetic bone that required conversion to other trajectory. This was performed in 1.8% of all screws.

No active bleeding as a result of vertebral artery injury was noted in any case neither Post operatively, there was clinical evidence of vertebral artery injury as all patients were observed for local neck hematomas, vertebrobasilar stroke; and for any further neurological deterioration. However, some co morbidities were encountered; fifteen patients experienced a persistent C5 nerve root pain with a satisfactory postoperative CT scan showing no violation by screws of the C4-C5 neural foramen except in one female patient that required revision and her symptoms improved after revision.

The cause of post operative C5 radicular pain is due to C5 nerve root traction the spinal cord is drifted backwards after laminectomy. There were six cases with superficial infection but no deep infection encountered. Only one case had CSF leak from the wound that treated successfully with reinforcement sutures and lumber drain for 3 days. No patient experienced screw or rod pullouts. However, late clinical DVT was observed only in four patients after a week of their surgery that required IVC filter insertion and anti coagulation therapy none of them developed pulmonary embolism clinically or wound. Only one female patient with cervical spine fracture died after 2 weeks of her surgery from massive pulmonary embolism. (Table 2).

**Table 2:** shows the post operative surgical complications of the lateral mass fixation

Complications: (number = 110 cases, 785 screws)	number	%
Vertebral artery injury	0	0

Root Injury secondary to screws	0	0

Dural tears	6	5.6

CSF leak	1	0.9

Superficial infection	6	5.6

Deep infection	0	0

Screw pullout or breakage(of 505 screws)	0	0

C5 root pain	15	13.6

Malposition that requires revision	1	0.9

DVT	4	6.7

Pulmonary embolism	1	0.9

Adjacent segment requiring surgery	0	0

Hematoma requiring evacuation	0	0

**Deaths**	**1**	**0.9**

The most common problem encountered was C5 radicular pain and incidental durotomy.

The results of the postoperative CT scan evaluation of screw position are shown in (Table 3). Significantly, 8 screws had violated the facet joint. 7 screws breached the foramen transversarium by less than 1m; anther 15 screws entered the neural foramen in variable levels, but one of them was significant at C5 level that ended with revision. No screw breached spinal canal.

**Table 3:** demonstrates the 405 lateral mass screw positions as evaluated by post op CT scan

Screw position assessed by CT scan	(number: 785 )	number%
appropriate obtain	765	97.5

Violation of foramen transversarium by less than 1 mm.	7	0.8

Breaching of neural foramen	5	0.6

Entering the spinal canal	0	0

**Violation facet joint **	**8**	**1.1**

In the long term follow up for 3 years no patient developed adjacent segment symptoms. Patients with C5 radicular pain revealed satisfactory response to facet joint block by using local steroid injection and amitryptiline pills (Table 4).

**Table 4:** reveals the analysis of long term follow up

Long term follow up and Outcomes	
Mean follow-up (mo)	20

Range of follow-up (mo)	3-36

Instrumentation failure	0

Adjacent segment disease	0

Late vascular or neural damage	

**Related to instrumentation**	**0**

There was no long term complication up to 36 months.

### Statistical analysis

In this study, age of patients with degenerative disc disease were approximated (P = .076) while it was variable for other pathologies. Patient's gender was male predominated (70%) (P = 0.311). The surgical principles for insertion of the lateral mass fixation was performed in the same manner. Additional surgical techniques were applied for vertebral body of the cervical spine tumours to achieve a gross total resection of the lesion and back up stabilization from anterior. In congenital anomalies of the cranio-cervical junction additional decompression of foramen magnum and incorporation of C2 pedicle was attained with satisfactory result. No neurovascular injury was observed. The vast majority of patients had a smooth operative and post operative courses. At the end of follow up for around 3 years the majority of survivors, basically the spondylotic, metabolic and congenital cases demonstrated a significant improvement. The co morbidities encountered in this study were not major and managed accordingly. Only one case developed a serious complication and she died of massive pulmonary embolism. Anther patient required adjustment of C5 screw because of persistent C5 radiculopathy and she improved after surgery.

This report documents that decompressive cervical laminectomy and lateral mass arthrodesis can be utilized for variable cervical spine pathologies with safety and efficiency. With emphasis on the use of Anderson- Sekhon trajectory as very reliable method and can provide adequate fixation with less violation of the adjacent structures.

## Discussion

Cervical spine is a place of many pathological lesions that could compromise its biomechanical stability. Restoration of stability may ultimately require fixation and placement of hard fixation devices. Posterior cervical spine stabilization is often administered to treat a variable cervical spine lesions that lead to spinal instability that include; cervical spondylotic degenerative disease, traumatic cervical spinal injury with and without fractures or with and without neurological deficits, metabolic -inflammatory lesions, primary and secondary neoplastic cervical spine lesions, infections, and in patients with previous wide cervical laminectomy without fixation. However, numerous surgical techniques and advances in spinal instrumentation have evolved over the last years. Lateral mass fixation has world widely gained popularity among spine surgeons with low morbidity and satisfactory outcome. Sekhon reported the largest series of subaxial lateral mass screw fixation with a total of 1024 screws and no related neuro-vascular injury observed [12,13].

Many screw entry points and directions have been described since this technique was first introduced, Roy-Camille advocated the entry point of the screw is the midpoint of the lateral mass and the direction of the screw is to be perpendicular to the posterior aspect of the cervical spine and 10^0 ^outward [1]. while Magerl proposed starting point is 2-3 mm medial and superior to the midpoint of the lateral mass and angling 30^0 ^superiorly and 25^0 ^laterally [14]. Anderson recommended that the drilling point is 1 mm medial to the midpoint of the lateral mass and that the screw be angled 30-40^0 ^up and 10^0 ^lateral [8]. An et al suggested angling 15-18^0^. superiorly and 30-33^0 ^laterally, with a starting point 1 mm medial to the center of the lateral mass [15]. Pait et al divided the lateral mass into four quadrants with the upper outer quadrant is the intention for screw insertion in this way its high likely to evade neurovascular injury [16]. finally, sekhon recommended that by using Anderson's starting point and then angling 25^0 ^laterally and superiorly; this way is safe and easily applied. In regards to the lateral mass of C7 it can be attained with a steeper course without need for C7 pedicle [13].

Frequent clinical and cadaver investigations have been done on lateral mass fixation. Focusing on various trajectories to achieve proper placement of the screw and to avoid neural and vascular damage. Ebraheim et al on his cadaver study revealed the foramen transverarium is located in line with the midpoint of the lateral mass. So, the direction of the screw is to be laterally to avoid entry into the vertebral foramen [17,18]. The work done by Xu et al reached that An technique is high likely to avoid neural damage compared to magerl and Anderson techniques [19,20]. However, the incidence of nerve root violation when Roy-Camille or Magerl, Sekhon trajectories used is around 3.6%; this is most likely because of the lengthy screw and more lateral trajectory [13,21,22].

In terms screw length, Roy-Camille et al recommended 14-17 mm [1]. An et al suggested a screw length of 11 mm is effective [15]. Sekhon suggested that a 14-mm screw is safe and efficient based on the fact that the average vertical distance between the posterior midpoint of the lateral mass and the vertebral foramen from C3 to C6 is approximately 9-12 mm [13]. As a result, insertion of a 14 mm screw obliquely should cross the lateral mass smoothly. In addition to that, a 14 mm screw can be bicorticate which adds further stability to the screw in place and causes no violation to the adjacent foramen, the Cadaveric studies of Heller et al concluded that bicorticate fixation with large diameter and non-self tapping screws had the utmost resistance to pullout [13,23-25].

In comparison with other fixation techniques such as cervical pedicle screws, lateral mass fixation is safer, has higher success rate and low co-morbidities. In early studies, the failure rate was higher patients who underwent screw/plate constructs compared with the newer polyaxial screw/rod systems. The former systems were semi constricted with no cross link; which augment the stability of the system. In general, the newer polyaxial screw/rod systems are more constrained and essentially avoid screw pullout [25-27].

## Conclusion

Wide Decompressive cervical Laminectomy with Lateral mass fixation by using Anderson - Sekhon trajectory is a safe and reliable surgical technique for posterior stabilization and proper for a wide range of cervical pathologies. With a long term follow up satisfactory results can be achieved. Neuro-vascular complication is usually low and is avoidable when using this trajectory.

## Competing interests

The authors declare that they have no competing interests.

## Authors' contributions

MMB: main surgeon, principal author; writing and design

ZAA: one of the main surgeons and second author, participated in the study design and analysis.

MMO: one of main surgeons, participated in the study design and analysis

TMQ: assistant surgeon and participated in patients' data collection

WFD: assistant surgeon and participated in patients' data collection

MHO assistant surgeon and participated in patients' data collection

AAM: assistant surgeon and participated in patients' data collection

All authors have read and approved the final manuscript.
